# Draft genome sequence of *Acinetobacter pittii* ST643 shared by cystic fibrosis patients

**DOI:** 10.1590/0074-02760160142

**Published:** 2016-09

**Authors:** Géssica A Rocha, Alex G Ferreira, Danielle F Lima, Robson S Leão, Ana Paula D Carvalho-Assef, Tânia W Folescu, Rodolpho M Albano, Elizabeth A Marques

**Affiliations:** 1 Universidade do Estado do Rio de Janeiro, Faculdade de Ciências Médicas, Departamento de Microbiologia, Imunologia e Parasitologia, Rio de Janeiro, RJ, Brasil; 2 Fundação Oswaldo Cruz, Instituto Oswaldo Cruz, Laboratório de Pesquisa em Infecção Hospitalar, Rio de Janeiro, RJ, Brasil; 3 Fundação Oswaldo Cruz, Instituto Nacional da Saúde da Mulher, da Criança e do Adolescente Fernandes Figueira, Departamento de Pneumologia Pediátrica, Rio de Janeiro, RJ, Brasil; 4 Universidade do Estado do Rio de Janeiro, Instituto de Biologia Roberto Alcântara Gomes, Departamento de Bioquímica, Rio de Janeiro, RJ, Brasil

**Keywords:** Acinetobacter pittii, cystic fibrosis, whole-genome sequencing

## Abstract

*Acinetobacter pittii* has emerged as an important hospital pathogen that is associated with outbreaks and drug resistance. In cystic fibrosis (CF) patients, the detection of *Acinetobacter* spp. is rare; however, we isolated the *A. pittii* sequence type ST643 in several Brazilian CF patients treated in the same centre. The current study describes the draft genome of *A. pittii* ST643.

*Acinetobacter* spp. are important nosocomial pathogens with *A. baumannii* being the most frequently observed species among clinical isolates (Peleg et al. 2008). However, in recent years, an increasing number of studies have highlighted the clinical significance of other *Acinetobacter* species, especially *A. pittii*, due to their association with resistance genes and hospital outbreaks (Teixeira et al. 2013, Yamamoto et al. 2013, Pagano et al. 2015). The mechanisms of resistance present in *Acinetobacter* spp. have been studied extensively, but few investigations have focused on understanding the virulence mechanisms that may contribute to the success of this species (Peleg et al. 2012).

Curiously, *Acinetobacter* spp. are not common pathogens in cystic fibrosis (CF), from which they are rarely isolated (Coenye et al. 2002). Nevertheless, we were successful in isolating *Acinetobacter* spp. strains from Brazilian CF patients. The identification of isolates to the species level by polymerase chain reaction (PCR) amplification and sequencing of a segment of the *rpoB* gene (Gundi et al. 2009) and by multilocus sequence typing (MLST) analysis (Diancourt et al. 2010) demonstrated that five strains (8863, 8876, 8893, 8895, 8941), isolated in the same period from different patients, belonged to sequence type ST643 of *A. pittii*. The disk diffusion method was performed to determine the antibiotic susceptibility of these isolates using the following antimicrobial agents: amikacin, ampicillin/sulbactam, cefepime, cefotaxime, ceftazidime, ciprofloxacin, gentamicin, imipenem, meropenem, piperacillin/tazobactam and tobramycin (CLSI 2015). Four strains were susceptible to all antimicrobials tested with the exception of cefotaxime. Strain 8876 was multidrug resistant (MDR), which included resistance to ciprofloxacin (quinolones), piperacillin/tazobactam (penicillin-type antibiotic/b-lactamase inhibitor) and cefotaxime (cephalosporins). This *A. pittii* 8876-ST643 MDR strain was selected for whole-genome sequencing.

This strain was isolated from the sputum of a Brazilian CF patient at Instituto Nacional da Saúde da Mulher, da Criança e do Adolescente Fernandes Figueira (IFF-FIOCRUZ), in 2009. A genomic library was constructed by transposon tagmentation with the Nextera XT DNA Sample Prep kit (Illumina Inc., USA). Two sequence runs were performed with the 500- and 300-cycle MiSeq Reagent v. 2 kits on a MiSeq benchtop sequencer (Illumina Inc. USA). A total of 1,445,561 and 1,232,439 paired-end reads for the 500- and 300-cycle kits, respectively, were obtained. Reads were corrected and assembled *de novo* using the genome assembler Spades 3.5 (Bankevich et al. 2012). The Rapid Annotation using System Technology (RAST) v. 2.0 server (http://rast.nmpdr.org), Center for Genomic Epidemiology website (ResFinder v. 2.1) (https://cge.cbs.dtu.dk//services/ResFinder/), Antibiotic Resistance Genes Database-ARDB (http://ardb.cbcb.umd.edu/) and BLASTP searches against GenBank were used for genome annotation. The resulting 147 scaffolds revealed a genome size of 4,014,293 bp and 74 rRNA genes. Of the 3,785 identified coding sequences, 49% were included in 458 subsystems by RAST, with the main subsystems being amino acids, amino acid derivatives and carbohydrates.

Annotation results indicate the presence of genes associated with virulence, such as those involved in adhesin biosynthesis (*pgaA*, *pgaB*, *pgaC* and *pgaD*), as well as genes involved in capsular polysaccharide biosynthesis and assembly (*wza*, *wzb* and *wzc*), toxin production (*dedA* and *colicinV*) and protein secretion systems (SST IV, SST V and SST VII). Genes associated with resistance to betalactams (*bla*_OXA417_ and *bla*_ADC-18_) and to quinolones (*cmeA*, *cmeB*, *cmeC* and *tetR*) were also identified. Although mutations in DNA gyrase and topoisomerase IV genes have also been associated with a decreased susceptibility to ciprofloxacin (Chiu et al. 2010), *A. pittii* 8876-ST643 did not show mutations within the QRDRs of *gyrA*, *gyrB*, *parC*, and *parE*. In addition to the aforementioned genes associated with the drug-resistance phenotype, genes involved with resistance to bacitracin (*bacA*), chloramphenicol (*ceoB*) and macrolides (*macA*, *macB* and *tolC*) were observed.

This genome sequence provides the basis for a future detailed genomic analysis that will certainly increase our understanding of the role of *A. pittii* in CF patients. This whole-genome shotgun project has been deposited at DDBJ/EMBL/GenBank under the accession LBCP00000000. The version described in this paper is version LBCP01000000.
